# Effects of Type 2 Diabetic Serum on Proliferation and Osteogenic Differentiation of Mesenchymal Stem Cells

**DOI:** 10.1155/2018/5765478

**Published:** 2018-06-05

**Authors:** Xiangqun Deng, Min Xu, Moyu Shen, Jinluo Cheng

**Affiliations:** ^1^Department of Endocrinology, Wuhan Third Hospital, Wuhan University, Wuhan 430062, China; ^2^Department of Endocrinology, Changzhou No. 2 People's Hospital, Nanjing Medical University, Changzhou 213003, China

## Abstract

Diabetic patients have an increased risk of osteoporosis-associated fractures. However, the results of most studies of the effects of diabetes on bone mass in patients with type 2 diabetes (T_2_DM) have been contradictory. To clarify these conflicting findings, we investigated the effects of diabetic serum on the proliferation and osteogenic differentiation of mesenchymal stem cells (MSCs). We used human sera from subjects with different levels of glycemic control to culture the MSCs and induce osteogenic differentiation. The rate of MSC proliferation differed when MSCs were cultured with sera from diabetic subjects with different levels of hyperglycemia. Hyperglycemic sera promoted MSC proliferation to some extent, but all the diabetic sera inhibited the differentiation of MSCs to osteoblasts. The effects of type 2 diabetic sera on the proliferation and osteogenic differentiation of MSCs are closely related to glycemic control. Our data demonstrate the importance of stratifying the study population according to glycemic control in clinical research into diabetic osteoporosis.

## 1. Introduction

Diabetes mellitus (DM) is a pandemic chronic metabolic disorder, with substantial morbidity and mortality, which is increasing in tandem with industrial development and an aging population. Growing evidence corroborates the notion that DM influences the skeletal metabolism, and patients with diabetes have an increased risk of osteoporosis-associated fractures. Many studies have demonstrated that patients with type 1 diabetes mellitus have reduced bone mineral density (BMD) and an increased risk of fracture [1–6]. However, although the relationship between type 2 diabetes mellitus (T_2_DM) and osteoporosis has been widely investigated, it remains controversial. A systematic review that analyzed 47 studies from the United States, Japan, China, Europe, and Africa [7] showed that all the studies reported conflicting effects of T_2_DM on bone mass. The majority of articles (26) showed increased BMD in T_2_DM, whereas 13 articles reported reduced BMD, and eight articles reported normal or no difference in bone mass. Several authors have reported elevated BMD among patients with T_2_DM in China [8], whereas other studies have reported reduced BMD [9–11]. These conflicting results regarding the influence of T_2_DM on BMD are probably partly attributable to differences in the sex, age, ethnicity, glycemic control, and body mass indices of the study subjects, but the specific mechanisms at the cellular and molecular levels require clarification.

The balance of osteoblasts and osteoclasts is an important factor in maintaining a normal bone mass. A reduction in osteoblast activity or an increase in osteoclast activity can influence the deposition and resorption of bone, inducing osteoporosis. Mesenchymal stem cells (MSCs) from the bone marrow are multipotent cells that can differentiate into multiple cell lineages, including osteoblasts, adipocytes, fibroblasts, myoblasts, and chondrocytes [12, 13]. Therefore, the quantity of MSCs and the proportion of MSCs that differentiate into osteoblast cells directly control the production of osteoblasts and, therefore, influence bone formation. Many studies have suggested that the differentiation of MSCs into a specific cell type should simultaneously inhibit their differentiation into other cell types [14–17]. The thiazolidinediones, peroxisome proliferator-activated receptor gamma agonists that are generally used in the treatment of T_2_DM, prevent osteoblast differentiation, thus increasing the differentiation of MSCs into adipocytes [18]. Consequently, an increase in the onset of osteoporosis is observed in diabetic patients treated with a thiazolidinedione, such as rosiglitazone. Osteoporosis is often accompanied by a histologically detectable increase in adipose tissue in the bone marrow, indicating the gradual replacement of the bone marrow by adipose tissue [19]. Therefore, a dysfunction in the proliferation and osteogenic differentiation of MSCs may be an important mechanism of osteoporosis in patients with T_2_DM.

In this study, to clarify the conflicting results regarding the influence of T_2_DM on BMD, we investigated the effects of T_2_DM on the proliferation and osteogenic differentiation of MSCs.

## 2. Materials and Methods

### 2.1. Instruments and Biochemical Reagents

A BD FACSCanto™ II flow cytometer (Becton Dickinson, Franklin Lakes, NJ, USA) was used to identify MSCs. The TC10™ Automated Cell Counter, iMark™ Microplate Absorbance Reader, CFX96™ Real-Time PCR System, PowerPac™ Electrophoresis System, and Gel DOC™ XR+ Imaging System were from Bio-Rad Laboratories, Inc. (Carlsbad, CA, USA). Percoll® cell-separating solution, used for gradient centrifugation, was from Santa Cruz Biotechnology, Inc. (Dallas, TX, USA). Gibco® cell culture reagents, such as penicillin, streptomycin, trypsin–EDTA (0.25%) with phenol red, fetal bovine serum (FBS), phosphate-buffered saline (PBS), minimum essential medium *α* (*α*-MEM), and Dulbecco's modified Eagle's medium (DMEM), were purchased from Thermo Fisher Scientific Inc. (Carlsbad,CA, USA). TRIzol Reagent and dimethyl sulfoxide (DMSO) were purchased from Invitrogen (Carlsbad, CA, USA). The RNA primers were synthesized by Shanghai Sangon Biotechnology Company Ltd (Shanghai, China). The iScript™ cDNA Synthesis Kit and SsoFast™ EvaGreen® Supermix for Quantitative Real-Time PCR were from Bio-Rad Laboratories, Inc. 3-(4,5-Dimethylthiazol-2-yl)-2,5-diphenyltetrazolium bromide (MTT), l-ascorbic acid 2-phosphate, l-glutamine, dexamethasone, *β*-glycerophosphate, and Alizarin red were purchased from Sigma (St. Louis, MO, USA).

Fluorescein isothiocyanate- (FITC-) conjugated mouse anti-human STRO-1 IgM antibody and the isotype control, FITC-conjugated mouse anti-human IgM antibody, were from Santa Cruz Biotechnology, Inc. RabMAbs® rabbit monoclonal antibodies directed against human RUNX2, osteopontin (OPN), osteocalcin (OCN), and glyceraldehyde 3-phosphate dehydrogenase (GAPDH) and a horseradish peroxidase- (HRP-) conjugated goat polyclonal secondary antibody directed against rabbit IgG H&L were purchased from Abcam (Hong Kong) Ltd. (New Territories, Hong Kong, China). Prestained marker, an SDS–polyacrylamide gel preparation kit, RIPA lysis buffer for western blotting, a BCA protein assay kit, and an enhanced chemiluminescence kit were supplied by Beyotime Biotechnology Company (Jiangsu, China). Other biochemical reagents were purchased from Shanghai Sangon Biotechnology Company Ltd.

### 2.2. Isolation, Culture, and Identification of Bone Marrow-Derived MSCs

All the protocols involving serum and cells that originated from humans, used either for experimentation or maintenance, were approved by the Ethics Committee of Changzhou Second People's Hospital Affiliated to Nanjing Medical University, Nanjing, China, and were performed in accordance with the Laboratory Guidelines of China. All the subjects gave their written informed consent in accordance with the Declaration of Helsinki.

Bone marrow aspirates (3–5 mL) were collected in heparin from four healthy volunteers and washed twice with PBS. The bone marrow cells were suspended and mixed with 5 mL of *α*-MEM containing 10% FBS. Each cell suspension was then added to an equal volume of 1.073 g/mL Percoll cell-separating solution for gradient centrifugation. After the bone marrow mononuclear cells were separated, they were placed in a 25 cm^2^ culture bottle and cultured (37°C, 5% CO_2_, saturated humidity) in *α*-MEM supplemented with 10% FBS, 10,000 units/mL penicillin, 10,000 *μ*g/mL streptomycin, and 0.2 mmol/L glutamine. The medium was changed every 3 days. The nonadherent cells were removed, and fresh general medium was added to allow further growth. After the adherent cells reached 80%–90% confluence, the culture medium was removed and the cells were washed three times with PBS. The adherent cells were detached with preheated 0.25% trypsin–EDTA and then subcultured in two new 75 cm^2^ culture bottles at a concentration of 8 × 10^3^ cells/cm^2^. The subcultured amplified cells were collected in the third generation and frozen for storage in cell-freezing medium, which contained *α*-MEM, 10% FBS, 10,000 units/mL penicillin, 10,000 *μ*g/mL streptomycin, and 10% DMSO. All the experiments were performed with cells from passage 4. The MSCs in the fourth generation were analyzed with flow cytometry for the expressions of the surface molecular marker STRO-1, as previously described [20].

### 2.3. Preparation of Human Blood Serum

To harvest the human blood sera, we collected blood samples from healthy and diabetic volunteers and classified them according to the level of glycosylated hemoglobin A1c (HbA1c), after each subject had given their written informed consent in accordance with the Declaration of Helsinki. The four human blood serum groups were divided according to HbA1c: <6.5%, 6.5%–8.0%, 8.0%–10%, and >10% HbA1c. 90 diabetic volunteers were enrolled in the study to supply their blood samples according to HbA1c level. Each subgroup included 30 diabetic volunteers, which their clinical parameters were matched for age, gender, and duration of T_2_DM. All diabetic volunteers received insulin therapy, and some were combined with metformin and/or acarbose in recent one year (Table 1). To avoid the influences of medicine on osteogenic differentiation of MSCs, all diabetic volunteers who received thiazolidinedione (rosiglitazone or pioglitazone) therapy were excluded. In addition, the diabetic patients with osteoporotic fracture were not enrolled in the study. 30 healthy volunteers with normal glycometabolic parameters were matched for age and gender to supply their blood samples with HbA1c < 6.5%. They were not being administered any medication. After the blood samples were collected, the sera were separated and mixed well according to HbA1c group. The serum mixtures were heated to 56°C in a water bath for about 30 min to inactivate the complement and then filter sterilized through a 0.22 *μ*m membrane. Each treated serum sample was split into aliquots for cryopreservation at −20°C.

### 2.4. MTT Cell Proliferation Assay of MSCs

Exponentially growing MSCs from passage 4 were collected for an MTT assay. The MSCs were seeded in 96-well plates at 10^3^ cells/well and cultured in 200 *μ*L of *α*-MEM supplemented with different blood sera for 2, 4, 5, 6, or 7 days. The medium was changed every 3 days. The 96-well plates were removed from the incubator at different time points, and 20 *μ*L of 0.5 mg/mL MTT solution was added to each well containing MSCs. The plate was centrifuged at low velocity for 5 min and incubated continuously for 4 h, and the medium removed. The plates were dried on paper towel, and 150 *μ*L of DMSO was added to each well to dissolve the cellular staining products. The optical density at a wavelength of 490 nm (OD_490_) was measured with a Bio-Rad iMark Microplate Absorbance Reader. The data derived from four healthy volunteers are shown as the mean ± SD of three independent wells. To determine the effects of T_2_DM on the proliferation of MSCs, we used cell culture solution *α*-MEM supplemented with different blood sera: 10% FBS, 5% FBS + 5% human blood serum or 10% human blood serum.

### 2.5. Induced Osteogenic Differentiation of MSCs

To induce the osteogenic differentiation of MSCs, MSCs (5000 cells/well) were plated in six-well plates. The osteoblast differentiation medium was high-glucose DMEM supplemented with 10% FBS, 50 mg/L ascorbic acid, 100 nM dexamethasone, and 10 mM *β*-glycerophosphate. The cells were cultured for 3 weeks and then stained with Alizarin red to detect the calcium phosphate deposits. To clarify the effects of diabetic serum on the osteogenic differentiation of MSCs, human sera with different concentrations of HbA1c were used to replace FBS in the MSC culture medium.

### 2.6. Quantitative Real-Time PCR

Total RNA was extracted from the cultured MSCs with TRIzol Reagent (Invitrogen), according to the manufacturer's instructions. The first-strand cDNA was synthesized from 1 *μ*g of total RNA in a 20 *μ*L reaction mixture using the iScript cDNA synthesis kit and then used as the template for PCR. Quantitative real-time PCR was performed with the Bio-Rad CFX96 Real-Time PCR System, with SsoFast EvaGreen Supermix, according to the manufacturer's instructions. GAPDH was used as the internal control. The PCR primers used in this study are listed in Table 2. The relative expression levels of the osteogenesis-associated genes were quantified with the comparative Ct(2^-∆∆Ct^) method [21].

### 2.7. Western Blotting Analysis

After osteogenic differentiation was induced in MSCs cultured for 21 days, the expression of osteogenesis-associated proteins RUNX2, OPN, and OCN, was analyzed with Western blotting. RabMAbs rabbit monoclonal antibodies directed against human RUNX2, OPN, OCN, and GAPDH were used to detect the corresponding proteins. The Western blotting analysis was performed as described previously [22]. Briefly, the MSCs were lysed in cold RIPA lysis buffer, and the lysed cell solutions were centrifuged at 12,000 ×g for 20 min at 4°C. The protein concentrations in the supernatants were determined with a BCA protein assay kit. Equivalent amounts of proteins were then separated with sodium dodecyl sulfate–polyacrylamide gel electrophoresis in a 10% acrylamide resolving gel. The separated proteins were electrotransferred onto polyvinylidene difluoride (PVDF) membrane, which were then blocked in TBS-T (1 × TBS, 0.1% Tween 20) containing 5% nonfat milk for 1 h at room temperature. The membrane was incubated at 4°C overnight with the RUNX2 antibody (1 : 1000), OPN antibody (1 : 1000), OCN antibody (1 : 1000), and GAPDH antibody (1 : 1000) as the internal reference. The PVDF membrane was then incubated with mouse anti-rabbit secondary antibody (1 : 20,000) for 2 h at room temperature and visualized with enhanced chemiluminescence reagent. After the membranes were exposed to Kodak film for 5–10 min, and the resulting images were quantitated with densitometry. The intensity of the bands was analyzed with the Image Lab™ software version 4.0 (Bio-Rad). The results were normalized to the density of the GAPDH bands.

### 2.8. Statistical Analysis

All the results were presented as mean ± SD and were analyzed with one-way ANOVA. The Student–Newman–Keuls multiple-range test was used to compare the results for two groups. *P* < 0.05 was considered statistically significant. To compare baseline and clinical characteristics of diabetic volunteers, we used Kruskal-Wallis tests for continuous variables and Fisher exact tests for discrete variables. All statistical analyses were performed with the SAS 8.1 software.

## 3. Results

### 3.1. MSC Morphology, Identification, and Osteogenic Differentiation

**Figure 1:**
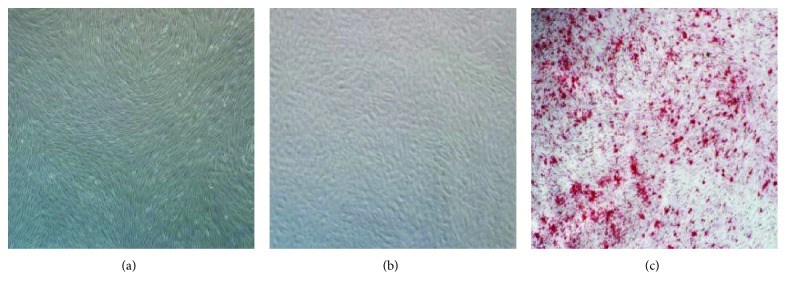
Morphology of primary and osteogenically differentiated MSCs (×40). (a) Primary cultured MSCs, slender, spindle-shaped, fibroblast-like cells with the typical spiral distribution. (b) MSCs after the induction of osteogenic differentiation; cells became round, with a cobble stone distribution. (c) After 21 days of osteogenic differentiation, large quantities of calcium nodules appeared in the MSCs. Alizarin red stained the calcium nodules scarlet.

After the bone marrow mononuclear cells were separated from the bone marrow with gradient centrifugation, they were seeded in a 25 cm^2^ culture flask. The primary MSCs had adhered to the culture flask wall 2 days after seeding. Several rounds of medium replacements and washing with PBS removed the nonadherent cells. The morphology of the adherent cells gradually changed, and they became slender, spindle-shaped, fibroblast-like cells from day 3 in culture. The cells proliferated rapidly and displayed clustering growth. The primary MSCs required culture for 2 weeks to reach 80%–90% confluence. However, the growth rate of the MSCs clearly increased after passaging, and the cells reached 100% confluence after only 1 week in culture. The typical spiral distribution of the MSCs is shown in Figure 1. The expression of STRO-1 is the best-known phenotypic characteristic of MSCs, and 99.6% of the cells tested positive for STRO-1. After osteogenic differentiation, the cell morphology of the MSCs changed markedly as they gradually became round, with a cobble stone distribution. Calcium nodules stained scarlet with Alizarin red, which are characteristic of osteogenic differentiation, were observed in the MSCs after osteogenic differentiation for 21 days (Figure 1). However, when we stained the cells with Alizarin red in different subgroups, we did not find the differences of staining images.

### 3.2. Effects of T_2_DM Sera on MSC Proliferation, Detected with an MTT Assay

**Figure 2:**
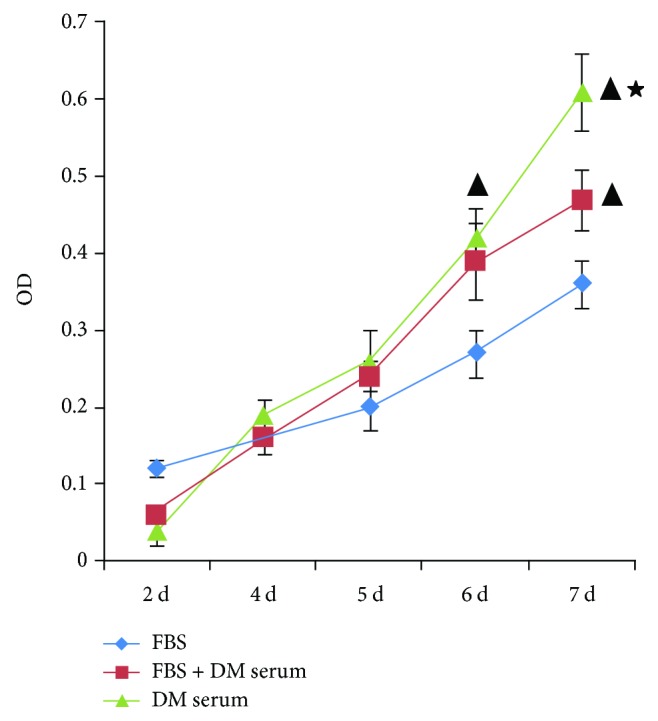
Proliferation of MSCs cultured with different amounts of serum, detected with an MTT assay. MCSs were divided into three groups: one group was cultured with 10% FBS; in one group, 10% FBS was replaced with 5% FBS + 5% diabetic serum; and in one group, 10% FBS was replaced with 10% diabetic serum. After 6 days in culture, the proliferation of the MSCs in the three groups differed significantly. All results were expressed as the mean ± SD and analyzed using one-way ANOVA. Student–Newman–Keuls multiple-range test was used to compare results between two groups: versus group FBS, ^▲^*P* < 0.01; versus group FBS + DM serum, ^★^*P* < 0.01. *n* = 4.

**Figure 3:**
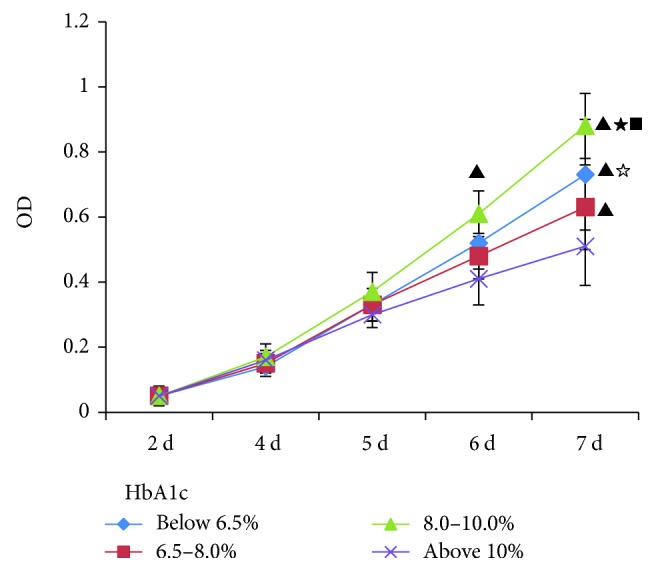
Proliferation of MSCs cultured with serum containing different levels of HbA1c, detected with an MTT assay. MCSs were divided into four groups, to which were added human blood sera with different levels of HbA1c: <6.5%, 6.5%–8.0%, 8.0%–10%, and >10%. After 6 days in culture, the proliferation of the MSCs differed significantly. Diabetic serum containing 8%–10% HbA1c stimulated cell proliferation most effectively. All results were expressed as the mean ± SD and analyzed using one-way ANOVA. Student–Newman–Keuls multiple-range test was used to compare results between two groups: versus group above 10%, ^▲^*P* < 0.01; versus group 6.5–8.0%, ^★^*P* < 0.01, ^☆^*P* < 0.05; versus group below 6.5%, ^■^*P* < 0.01. *n* = 4.

To analyze the proliferation of the MSCs, they were seeded in 96-well plates at 10^3^ cells/well, the optimal density for the MTT assay. When cultured with FBS for about 7 days, the cells in the 96-well plates reached 100% confluence. The 10% FBS was then replaced partly or fully with T_2_DM serum: either 5% FBS + 5% diabetic serum or 10% diabetic serum. Surprisingly, the diabetic serum significantly increased the proliferation of the MSCs (Figure 2). The diabetic sera were divided into different grades with different concentrations of HbA1c and then added to the culture medium for MSCs. Compared with FBS treatment alone, human serum (including normal and diabetic serum) clearly increased cell proliferation. The sera from diabetic patients in which HbA1c was 8%–10% stimulated cell proliferation more effectively than normal serum (<6.5% HbA1c). In contrast, serum from diabetic patients in which HbA1c was >10% stimulated cell proliferation less effectively than normal serum (<6.5% HbA1c) (Figure 3).

### 3.3. Effects of Diabetic Serum on mRNA Expression of Osteogenic Differentiation-Associated Genes

**Figure 4:**
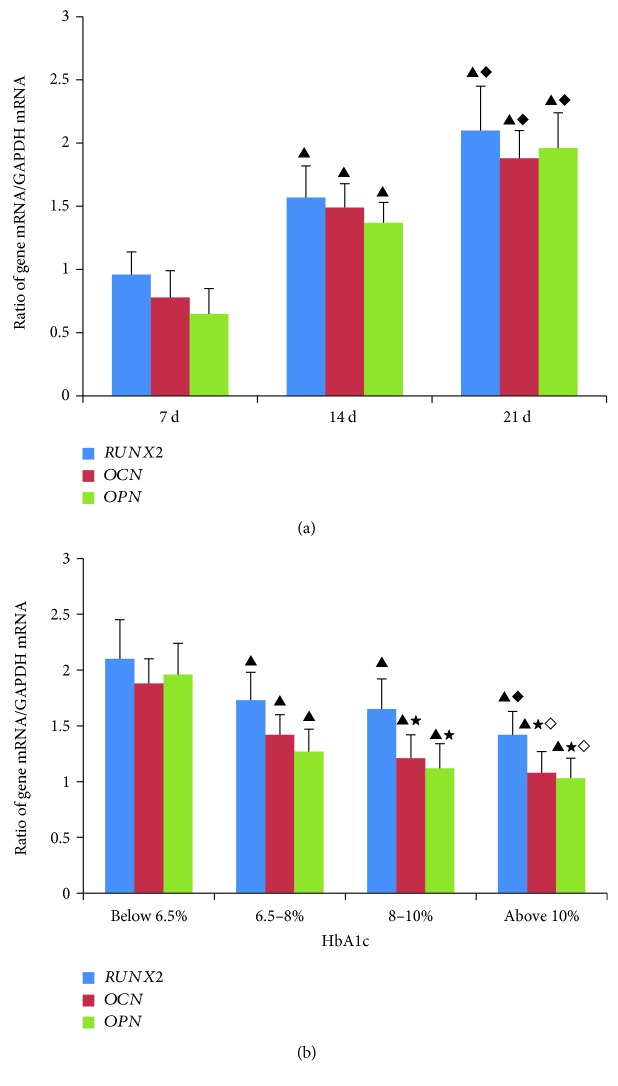
(a) mRNA expression of osteogenic differentiation-associated genes in different periods of culture, detected with quantitative real-time PCR. Osteogenic differentiation-related genes *RUNX2*, *OPN*, and *OCN* were detected after MSCs were cultured for different periods. mRNA expression increased gradually as the culture time increased. All results were expressed as the mean ± SD and analyzed using one-way ANOVA. Student–Newman–Keuls multiple-range test was used to compare results between two groups: versus culture 7 d, ^▲^*P* < 0.01; versus culture 14 d, ^♦^*P* < 0.01. *n* = 4. (b) mRNA expression of osteogenic differentiation-associated genes in MSCs cultured with human sera. Osteogenic differentiation of MSCs was induced by culture with human serum. Cells were cultured in sera containing different concentrations of HbA1c: <6.5%, 6.5%–8.0%, 8.0%–10%, or >10%. mRNA expression was detected when the MSCs had been cultured for about 21 days. Diabetic sera inhibited the expression of osteogenic differentiation-associated genes. All results were expressed as the mean ± SD and analyzed using one-way ANOVA. Student–Newman–Keuls multiple-range test was used to compare results between two groups: versus group below 6.5%, ^▲^*P* < 0.01; versus group 6.5–8%, ^★^*P* < 0.01; versus group 8–10%, ^♦^*P* < 0.01, ^◊^*P* < 0.05. *n* = 4.

First, we induced the osteogenic differentiation of MSCs in FBS-supplemented culture medium. The mRNA expression of the osteogenic differentiation factors RUNX2, OPN, and OCN increased gradually as the culture time increased. When MSCs had been cultured for about 21 days, calcium nodules were observed and the mRNA expression of the osteogenic differentiation-associated genes had increased significantly, suggesting that the MSCs had differentiated into osteoblasts (Figure 4(a)). The serum used to induce osteogenic differentiation was then replaced with human serum. All the diabetic sera inhibited the mRNA expression of the osteogenic differentiation-associated genes relative to their expression in MSCs treated with normal human serum. Sera with higher concentrations HbA1c more effectively reduced the expression of these genes after the MSCs had been cultured for about 21 days (Figure 4(b)).

### 3.4. Expression of Osteogenic Differentiation-Associated Proteins

**Figure 5:**
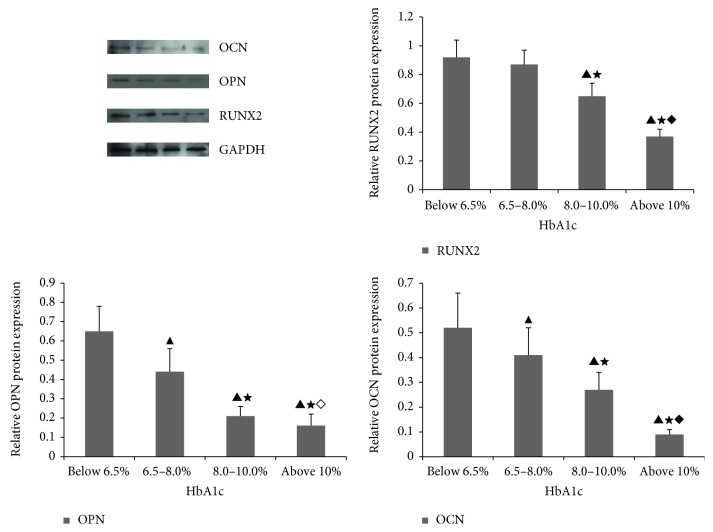
Expression of osteogenic differentiation-associated proteins. Osteogenic differentiation of MSCs was induced by culture with human serum. Human diabetic sera were divided into four groups according to HbA1c: <6.5%, 6.5%–8.0%, 8.0%–10%, and >10%. Protein expression was detected after 21 days in culture. All results were expressed as the mean ± SD and analyzed using one-way ANOVA. Student–Newman–Keuls multiple-range test was used to compare results between two groups: versus group below 6.5%, ^▲^*P* < 0.01; versus group 6.5–8.0%, ^★^*P* < 0.01; versus group 8.0–10%, ^♦^*P* < 0.01, ^◊^*P* < 0.05. *n* = 4.

We investigated the expression of osteogenic differentiation-associated proteins when MSCs were cultured with human serum containing different levels of HbA1c to induce the osteogenic differentiation of MSCs. Protein expression was detected after 21 days in culture. Compared with normal serum (<6.5% HbA1c), the expression of proteins RUNX2, OPN, and OCN clearly decreased as the HbA1c concentration increased (Figure 5).

## 4. Discussion

The dysfunction in the proliferation and osteogenic differentiation of MSCs may be an important mechanism of osteoporosis in patients with T_2_DM. We used human sera from subjects with different levels of glycemic control to culture the MSCs and induce osteogenic differentiation. We found that the rate of MSC proliferation differed when MSCs were cultured with sera from diabetic subjects with different levels of hyperglycemia, but all the diabetic sera inhibited the differentiation of MSCs to osteoblasts. The effects of type 2 diabetic sera on the proliferation and osteogenic differentiation of MSCs are closely related to glycemic control.

At present, the development of osteoporosis is considered to be associated with factors such as endocrine levels, genes, nutrition, vitamins, and lifestyle [23–29]. However, the specific causes of osteoporosis are not yet completely understood. The diabetic population is a high-risk group for osteoporosis, but even in developed countries, there have been few systematic studies of diabetes-induced osteoporosis [30]. Among the many causes of osteoporosis, the suppression of MSC differentiation to osteoblasts may be the most basic and direct mechanism. Therefore, studying the proliferation and osteogenic differentiation of MSCs in diabetic patients may be an effective way to clarify at the cellular level the so far discrepant findings on the influence of T_2_DM on BMD.

Several studies [31, 32] have suggested that FBS plays an important role in the culture of MSCs. The equal quantity MSCs can generate greater quantitative difference colony-forming units of fibroblasts (CFU-Fs) and different quantity cells in each CFU-Fs when different levels of FBS were used to culture the MSCs. In the present study, we examined the effects of human diabetic serum on MSC proliferation and osteogenic differentiation. First, we isolated MSCs from the bone marrow of healthy volunteers and grew them in primary culture. We then used diabetic sera to partly or completely replace FBS in the MSC culture medium. The diabetic serum stimulated MSC proliferation significantly more effectively than FBS. Because the diabetic serum we used was a mixture of sera from different T_2_DM patients, it did not reflect the effects of diabetic sera from patients with different degrees of glycemic control. Because HbA1c is the gold standard for assessing glycemic control [33, 34], we mixed the human blood sera according to their HbA1c concentrations: <6.5%, 6.5%–8.0%, 8.0%–10%, or >10%. The MSC proliferation rate differed when the MSCs were cultured with human blood sera containing different levels of HbA1c. The MSC proliferation rate in the 8%–10% HbA1c group was significantly higher that than in the normal control group (<6.5% HbA1c), whereas the MSC proliferation rate in the >10% HbA1c group was significantly reduced. These results suggest that a certain degree of hyperglycemia promotes MSC proliferation, whereas high HbA1c levels (>10%), which reflect poor glycemic control, significantly inhibit MSC proliferation.

The osteogenic differentiation of MSCs, which differs from their proliferation, displayed consistent results when cultured with different levels of HbA1c. During the induction of MSC differentiation to osteoblasts, all the diabetic sera inhibited the expression of osteogenic differentiation-associated genes at the mRNA and protein levels, suggesting that diabetic serum inhibits the differentiation of MSCs to osteoblasts. Furthermore, higher levels of HbA1c more effectively inhibited the osteogenic differentiation of the MSCs. This suggests that poor glycemic control has a large negative effect on the differentiation of MSCs to osteoblasts.

At the cellular level, the abnormal proliferation and osteogenic differentiation of MSCs induce osteoporosis, and osteoporosis is the combined effect of defects in the proliferation and osteogenic differentiation of MSCs [35, 36]. The present study may partly explain previous conflicting results regarding the influence of T_2_DM on BMD. In diabetic patients, a moderate increase in hyperglycemia significantly promotes MSC proliferation, but slightly inhibits their differentiation to osteoblasts, and these combined effects on MSC proliferation and osteogenic differentiation may increase BMD. However, with poorly controlled hyperglycemia, when HbA1c exceeds 10%, for example, BMD is reduced because both the proliferation and osteogenic differentiation of MSCs are inhibited. Our results show that the BMD of patients with T_2_DM is closely related to their long-term glycemic control and demonstrate the importance of stratifying study subjects according to their glycemic control in clinical investigations of diabetic osteoporosis.

However, we only investigated the effects of T_2_DM sera on the proliferation and osteogenic differentiation of MSCs in this study. Diabetic serum is a very complex mixture, and the key factors in the serum that affect MSCs are not yet clear. Nevertheless, the use of human serum for in vitro assays used by D'Addio et al. [37] is still a valuable tool to test the effect of unknown circulating factors. Furthermore, diabetic T_2_DM MSCs themselves may be abnormal in their capacities for proliferation and osteogenic differentiation. Therefore, further studies are required to clarify these issues.

## 5. Conclusions

This study demonstrates that a moderate increase in hyperglycemia promotes the proliferation of MSCs, whereas poorly controlled hyperglycemia inhibits them. Hyperglycemia also inhibits the osteogenic differentiation of MSCs and the extent of this inhibition is also closely related to glycemic control. Our findings demonstrate the importance of stratifying study subjects according to their glycemic control in clinical research into diabetic osteoporosis. This study partly clarifies the previous conflicting findings regarding the influence of T_2_DM on BMD.

## Figures and Tables

**Table 1:** Clinical characteristics of diabetic volunteers.

Variable	HbA1c 6.5%–8.0%	HbA1c 8.0%–10%	HbA1c above 10%	*P* value
Number of diabetic patients	30	30	30	
Sex				0.87
Male	18 (60)	17 (57)	18 (60)	
Female	12 (40)	13 (43)	12 (40)	
Age (years)	48.2 ± 8	48.6 ± 9	48.4 ± 10	0.92
Duration of diabetes (years)	12.1 ± 5	12.6 ± 7	13.2 ± 5	0.68
Diabetes therapy				
Insulin alone	20 (67)	18 (60)	18 (60)	0.92
Insulin and metformin	2 (7)	2 (7)	2 (7)	>0.99
Insulin and acarbose	1 (3)	1 (3)	2 (7)	0.94
Insulin and metformin and acarbose	7 (23)	9 (30)	8 (26)	0.25

Data were presented as means ± SD or *n* (%).

**Table 2:** Primer sequences and product lengths for real-time PCR.

Gene	Primer sequences	Product (bp)
GAPDH-F	CTCTGCTCCTCCTGTTCGACA	112
GAPDH-R	ACGACCAAATCCGTTGACTC	
RUNX2-F	CCGGAATGCCTCTGCTGTTATGA	238
RUNX2-R	ACTGAGGCGGTCAGAGAACAAACT	
OPN-F	ACAGCCACAAGCAGTCCAGATT	109
OPN-R	TGGAATTCACGGCTGACTTTG	
OCN-F	GACTGTGACGAGTTGGCTGA	119
OCN-R	CTGGAGAGGAGCAGAACTGG	

## References

[1] Auwerx J., Dequeker J., Bouillon R., Geusens P., Nijs J. (1988). Mineral metabolism and bone mass at peripheral and axial skeleton in diabetes mellitus. *Diabetes*.

[2] Gunczler P., Lanes R., Paz-Martinez V. (1998). Decreased lumbar spine bone mass and low bone turnover in children and adolescents with insulin dependent diabetes mellitus followed longitudinally. *Journal of Pediatric Endocrinology & Metabolism*.

[3] Lettgen B., Hauffa B., Möhlmann C., Jeken C., Reiners C. (1995). Bone mineral density in children and adolescents with juvenile diabetes: selective measurement of bone mineral density of trabecular and cortical bone using peripheral quantitative computed tomography. *Hormone Research*.

[4] Leon M., Larrodera L., Lledo G., Hawkins F. (1989). Study of bone loss in diabetes mellitus type 1. *Diabetes Research and Clinical Practice*.

[5] Forst T., Pfützner A., Kann P. (1995). Peripheral osteopenia in adult patients with insulin-dependent diabetes mellitus. *Diabetic Medicine*.

[6] Mastrandrea L. D., Wactawski-Wende J., Donahue R. P., Hovey K. M., Clark A., Quattrin T. (2008). Young women with type 1 diabetes have lower bone mineral density that persists over time. *Diabetes Care*.

[7] Abdulameer S. A., Sulaiman S. A., Hassali M. A., Subramaniam K., Sahib M. N. (2012). Osteoporosis and type 2 diabetes mellitus: what do we know, and what we can do?. *Patient Preference and Adherence*.

[8] Shan P. F., Wu X. P., Zhang H. (2009). Bone mineral density and its relationship with body mass index in postmenopausal women with type 2 diabetes mellitus in mainland China. *Journal of Bone and Mineral Metabolism*.

[9] Hsu Y., Chen C., Feng F. (2004). Major determinants of bone mineral density (BMD) at multiple skeletal sites in Chinese. *Journal of Bone and Mineral Research*.

[10] Xu L., Cheng M., Liu X., Shan P., Gao H. (2007). Bone mineral density and its related factors in elderly male Chinese patients with type 2 diabetes. *Archives of Medical Research*.

[11] Zhou Y., Li Y., Zhang D., Wang J., Yang H. (2010). Prevalence and predictors of osteopenia and osteoporosis in postmenopausal Chinese women with type 2 diabetes. *Diabetes Research and Clinical Practice*.

[12] Kilian K. A., Bugarija B., Lahn B. T., Mrksich M. (2010). Geometric cues for directing the differentiation of mesenchymal stem cells. *Proceedings of the National Academy of Sciences of the United States of America*.

[13] Valtieri M., Sorrentino A. (2008). The mesenchymal stromal cell contribution to homeostasis. *Journal of Cellular Physiology*.

[14] Knothe Tate M. L., Falls T. D., McBride S. H., Atit R., Knothe U. R. (2008). Mechanical modulation of osteochondroprogenitor cell fate. *The International Journal of Biochemistry & Cell Biology*.

[15] Heino T. J., Hentunen T. A. (2008). Differentiation of osteblasts and osteocytes from mesenchymal stem cells. *Current Stem Cell Research & Therapy*.

[16] Zhao L., Hantash B. M. (2011). Chapter seven—TGF-*β*1 regulates differentiation of bone marrow mesenchymal stem cells. *Vitamins & Hormones*.

[17] Li J., Zhang N., Huang X. (2013). Dexamethasone shifts bone marrow stromal cells from osteoblasts to adipocytes by C/EBPalpha promoter methylation. *Cell Death & Disease*.

[18] Jeon M. J., Kim J. A., Kwon S. H. (2003). Activation of peroxisome proliferator-activated receptor-gamma inhibits the Runx2-mediated transcription of osteocalcin in osteoblasts. *Journal of Biological Chemistry*.

[19] Kawai M., de Paula F. J. A., Rosen C. J. (2012). New insights into osteoporosis: the bone–fat connection. *Journal of Internal Medicine*.

[20] Shen Y., Wang W., Li X., Liu Z., Markel D. C., Ren W. (2012). Impacts of age and gender on bone marrow profiles of BMP7, BMPRs and Stro-1^+^ cells in patients with total hip replacement. *International Orthopaedics*.

[21] Pfaffl M. W. (2001). A new mathematical model for relative quantification in real-time RT–PCR. *Nucleic Acids Research*.

[22] Mabley J. G., Belin V., John N., Green I. C. (1997). Insulin-like growth factor I reverses interleukin-1*β* inhibition of insulin secretion, induction of nitric oxide synthase and cytokine-mediated apoptosis in rat islets of Langerhans. *FEBS Letters*.

[23] Raisz L. G. (2005). Pathogenesis of osteoporosis: concepts, conflicts, and prospects. *The Journal of Clinical Investigation*.

[24] Kuchuk N. O., van Schoor N. M., Pluijm S. M., Smit J. H., de Ronde W., Lips P. (2007). The association of sex hormone levels with quantitative ultrasound, bone mineral density, bone turnover and osteoporotic fractures in older men and women. *Clinical Endocrinology*.

[25] Li W. F., Hou S. X., Yu B., Li M. M., Férec C., Chen J. M. (2010). Genetics of osteoporosis: accelerating pace in gene identification and validation. *Human Genetics*.

[26] Ferrari S. (2008). Human genetics of osteoporosis. *Best Practice & Research Clinical Endocrinology & Metabolism*.

[27] Masse P. G., Jougleux J. L., Tranchant C., Dosy J., Caissie M., Coburn S. P. (2010). Enhancement of calcium/vitamin d supplement efficacy by administering concomitantly three key nutrients essential to bone collagen matrix for the treatment of osteopenia in middle-aged women: a one-year follow-up. *Journal of Clinical Biochemistry and Nutrition*.

[28] Tucker K. L. (2009). Osteoporosis prevention and nutrition. *Current Osteoporosis Reports*.

[29] Ali N. S., Shonk C., El-Sayed M. S. (2009). Bone health in men: influencing factors. *American Journal of Health Behavior*.

[30] Chau D. L., Edelman S. V., Chandran M. (2003). Osteoporosis and diabetes. *Current Diabetes Reports*.

[31] Lennon D. P., Haynesworth S. E., Bruder S. P., Jaiswal N., Caplan A. I. (1996). Human and animal mesenchymal progenitor cells from bone marrow: identification of serum for optimal selection and proliferation. *In Vitro Cellular & Developmental Biology - Animal*.

[32] Kuznetsov S. A., Mankani M. H., Bianco P., Robey P. G. (2009). Enumeration of the colony-forming units–fibroblast from mouse and human bone marrow in normal and pathological conditions. *Stem Cell Research*.

[33] Sacks D. B. (2012). Measurement of hemoglobin A_1c_: a new twist on the path to harmony. *Diabetes Care*.

[34] Sherwani S. I., Khan H. A., Ekhzaimy A., Masood A., Sakharkar M. K. (2016). Significance of HbA1c test in diagnosis and prognosis of diabetic patients. *Biomarker Insights*.

[35] Luu Y. K., Capilla E., Rosen C. J. (2009). Mechanical stimulation of mesenchymal stem cell proliferation and differentiation promotes osteogenesis while preventing dietary-induced obesity. *Journal of Bone and Mineral Research*.

[36] Sun N., Yang L., Li Y. (2013). Effect of advanced oxidation protein products on the proliferation and osteogenic differentiation of rat mesenchymal stem cells. *International Journal of Molecular Medicine*.

[37] D'Addio F., La Rosa S., Maestroni A. (2015). Circulating IGF-I and IGFBP3 levels control human colonic stem cell function and are disrupted in diabetic enteropathy. *Cell Stem Cell*.

